# Association between Early Neuroretinal Dysfunction and Peripheral Motor Unit Loss in Patients with Type 1 Diabetes Mellitus

**DOI:** 10.1155/2018/9763507

**Published:** 2018-10-04

**Authors:** Fabiana Picconi, Giorgia Mataluni, Lucia Ziccardi, Mariacristina Parravano, Antonio Di Renzo, Dorina Ylli, Patrizio Pasqualetti, Valeria Studer, Laura Chioma, Girolama Alessandra Marfia, Simona Frontoni

**Affiliations:** ^1^Unit of Endocrinology, Diabetes and Metabolism, S. Giovanni Calibita Fatebenefratelli Hospital, Department of Systems Medicine, University of Rome Tor Vergata, Rome, Italy; ^2^Unit of Disimmune Neuropathies, Department of Systems Medicine, University of Rome Tor Vergata, Rome, Italy; ^3^IRCCS-G.B. Bietti Foundation, Rome, Italy; ^4^Division of Endocrinology MedStar Washington Hospital Center, MedStar Health Research Institute, Washington, DC, USA; ^5^Fatebenefratelli Foundation for Health Research and Education, AFaR Division, Rome, Italy; ^6^Neuroimmunology and Neuromuscolar Diseases Unit, Foundation IRCCS Neurological Institute Carlo Besta, Milan, Italy

## Abstract

**Objectives:**

It has been already confirmed that retinal neurodegeneration has a predictive value in the development of microvascular alterations in diabetic retinopathy. However, no data are available on the association between neuroretinal dysfunction and peripheral motor unit loss. Our study, therefore, was aimed at investigating the hypothesis that retinal neurodegeneration could be considered an early marker of diabetic peripheral neuropathy (DPN).

**Methods:**

20 T1DM patients with no symptoms/signs of peripheral polyneuropathy, without DR or with very mild nonproliferative DR, and 14 healthy controls (C) age- and gender-matched were enrolled. The following electrophysiological tests were performed: standard nerve conduction studies (NCS) and incremental motor unit number estimation (MUNE) from the abductor hallux (AH) and abductor digiti minimi (ADM). Neuroretinal function was studied by multifocal electroretinogram (MfERG) recordings, measuring response amplitude density (RAD) and implicit time (IT) from rings and sectors of superior (S)/inferior (I)/temporal (T)/nasal (N) macular sectors up to 10 degrees of foveal eccentricity.

**Results:**

MfERG RADs from rings and sectors were significantly reduced in T1DM (*p* < 0.05) vs. C. ADM MUNE and AH MUNE were significantly decreased in T1DM (*p* = 0.039 and *p* < 0.0001, respectively) vs. C. A positive correlation between mean MfERG RADs from the central 5 degrees of the four (S, I, T, and N) macular sectors and lower limb motor unit number (*r* = 0.50, *p* = 0.041; *r* = 0.64, *p* = 0.005; *r* = 0.64, *p* = 0.006; and *r* = 0.61, *p* = 0.010, respectively) was observed in T1DM patients. No abnormalities of NCS were found in any subject.

**Conclusions:**

The motor unit loss on the one hand and neuroretinal dysfunction on the other hand are already present in T1DM patients without DPN. The relationship between neuroretinal dysfunction and motor unit decline supports the hypothesis that neuroretina may represent a potential “window” to track the early neurogenic damage in diabetes.

## 1. Introduction

Diabetic peripheral neuropathy (DPN) is one of the most debilitating complications of diabetes mellitus (DM), determining sensory loss and neuropathic pain and late weakness [1]. The diagnosis of diabetic neuropathy is also based on the use of neurophysiologic techniques and the evaluation of advanced signs and symptoms. Moreover, the ability of standard electrophysiological techniques (nerve conduction studies and electromyographic evaluation) to identify neuronal dysfunction is confined to overt neuropathic damage. On the other hand, the new procedures, such as skin biopsy, showed to be helpful in detecting neuropathy in the early and subclinical stages but they are still demanding because they are invasive, require expensive laboratory equipment and trained personnel, and still have limited reproducibility [2]. A painless, noninvasive, cost-effective, and clinically available tool is therefore necessary, for the early detection, staging, and monitoring of peripheral diabetic neuropathy [3].

Recently, emerging evidence shows that the neuropathic process, contrary to the conventional view, is not only confined to the peripheral sensory nerves but also involved the whole nervous system, thus suggesting the possible identification of new observational “windows” of the neurodegenerative process [4]. Particularly, in few studies on animal models and in humans, an early impairment of the motor nerves has also been demonstrated [5, 6]. These alterations are likely associated to a “dying back” of motor nerve terminals, similar to the death process that occurs in sensory epidermal nerve fibers [6].

On the other hand, the process of retinal neurodegeneration is known to precede vascular damage, representing so far an early marker of diabetic retinopathy (DR) [7–9]. However, despite its prominence at clinical examination, vasculature makes up less than 5% of the retinal mass, so that the retina can be more appropriately considered as a vascularized neuronal tissue [10, 11]. Therefore, since hyperglycemia adversely affects the entire neurosensory retina, by accelerating neuronal apoptosis or altering metabolism of neuroretinal supporting cells [11, 12], some authors suggest to consider diabetic retinopathy, as a neuropathy that affects the retinal parenchyma, similar to peripheral diabetic neuropathy [13]. The aim of this observational study is to explore neuronal damage in type 1 diabetes mellitus through new clinically accessible “windows” as the retinal nerve tissue and peripheral motor units and to analyze if their dysfunctions are potential early markers of neurodegeneration in patients without peripheral diabetic neuropathy.

## 2. Materials and Methods

We followed the methods of Picconi et al. [14]. From the Unit of Endocrinology, Diabetes and Metabolism, S. Giovanni Calibita Fatebenefratelli Hospital of Rome, we recruited 20 patients with type 1 DM (T1DM). 14 healthy participants, without history of ocular disease and no family history of glaucoma or any relevant systemic disease, were enrolled as control group (C), from the medical staff of the Medical Retina Unit, G.B. Bietti Eye Foundation-IRCCS, Rome. Inclusion criteria for the type 1 DM patients were (1) documented diagnosis of type 1 DM, according to ADA criteria [15]; (2) age between 18 and 75 years; (3) treated with continuous subcutaneous insulin infusion or with multiple daily insulin injections; and (4) no signs of retinal vasculopathy (noDR) or very mild nonproliferative diabetic retinopathy (NPDR). Exclusion criteria were (1) symptomatic diabetic polyneuropathy not even with positive sensory symptoms such as pain, burning, paresthesia, or prickling; (2) history of possible confounding diseases (alcohol abuse, vitamin deficiency, malignancy treated with chemotherapy agents, central nervous system diseases, entrapment mononeuropathies, and cervical or lumbosacral radiculopathies); (3) a Michigan Neuropathy Screening Instrument [16] score equal to or greater than 2 points; (4) microalbuminuria (urinary albumin/creatinine ratio > 30 mg/g); (5) spherical refractive error > ±3 diopters, astigmatism (cyl) > ±2 diopters, active or past retinal pathologies, diagnosis of glaucoma or ocular hypertension, and opacities of optical media that could influence functional and structural retinal testing; and (6) history of ocular surgery. This study complied with the principles of the Declaration of Helsinki. All subjects gave their written informed consent.

All subjects underwent a general medical examination and anthropometric parameters. After an overnight fast, blood and urine samples were obtained for the determination of laboratory measurements. Each person underwent a complete ophthalmological examination, with determination of best-corrected visual acuity, anterior segment examination, fundus photography, and multifocal electroretinogram (MfERG) recordings. Neurological evaluation was performed at Disimmune Neuropathies Unit, Policlinico Tor Vergata of Rome. All patients underwent electrophysiological examination including bilateral standard sensory motor nerve conduction studies (NCS) and motor unit number estimation (MUNE). Extensive clinical neurological evaluation was performed; strength was assessed by means of Medical Research Council (MRC) sum score (with a maximum score of 60/60 indicating full strength) [17]. The MRC score of the muscles from which MUNE was derived (abductor digiti minimi (ADM) and abductor hallux (AH)) was also calculated.

### 2.1. MUNE Evaluation

MUNE is an electrophysiological method that can be used to determine the approximate functioning number of motor neuron units or axons innervating a single muscle. In addition, MUNE methods provide a means of measuring motor unit size, enabling tracking of both loss of motor units and the compensatory phenomenon of collateral reinnervation, and have the advantage of measuring the severity of nerve injury in neuropathy with retained CMAP amplitude; MUNE is already used in neuromuscular disorders such as amyotrophic lateral sclerosis, spinal muscular atrophy, and neuropathies to monitor neuronal loss [18, 19].

Recordings are performed using Medtronic Keypoint EMG equipment (Skovlunde Denmark). Limb temperature is maintained between 32 and 34°C by a heating lamp. Filter settings are 2 Hz/10 kHz. The maximal compound motor action potential (CMAP) is obtained by supramaximal stimulation of the peroneal nerve at the lower limbs and of the ulnar nerve at the upper limbs, with constant current square waves at the fibular head site and from the wrist site, respectively. Measurement of the CMAP negative peak area (from the onset of the first negative peak to the first crossing of the baseline) is preferred to peak-to-peak amplitude or negative peak amplitude measurements, as it minimizes the cancellation error [20] and better considers collateral reinnervation phenomena. Recordings are made from the abductor digiti minimi (ADM) for the ulnar nerve and from the abductor hallux (AH) for the tibial posterior nerve, with a single active surface electrode over the belly of the muscle, an inactive electrode over the tendon, and a ground surface electrode positioned between the recording site and stimulating site, similar to the arrangement for routine NCS. In this study, we determined the MUNE of ADM and AH using the manual incremental method [21], in which the assumption is made that each small, stepwise increase in CMAP amplitude with slight increments of stimulus intensity represented the addition of another single motor unit potential (SMUP) to the growing waveform [22]. A series of consecutive stimuli of progressively higher intensity are therefore applied at the stimulation site to obtain 10 distinct SMUP, elicited in an all-or-nothing manner. The area of each increment is measured and averaged to get the average SMUP area for that nerve and muscle. As the maximal CMAP area represents the total motor unit population firing together, dividing the maximal CMAP area by the average SMUP area yields an estimate of the number of motor units within that nerve. MUNE value is therefore expressed as maximal CMAP area/average SMUP area. In addition to an estimate of motor unit number, the average SMUP size obtained with these methods is also calculated, in order to quantify the extent of collateral reinnervation.

### 2.2. MfERG Recordings

VERIS Clinic™ 4.9 (Electro-Diagnostic Imaging, San Mateo, California, USA) was used for MfERG assessment using our previously published method [23–25]. The multifocal stimulus, consisting of 61 scaled hexagons, was displayed on a high-resolution, black-and-white monitor (size: 30 cm width and 30 cm height) with a frame rate of 75 Hz. The array of hexagons subtended 20 degrees of visual field. Each hexagon was independently alternated between black (1 cd/m^2^) and white (200 cd/m^2^) according to a binary *m*-sequence. This resulted in a contrast of 99%. In all eyes, MfERGs were binocularly recorded in the presence of pupils that were maximally pharmacologically dilated with 1% tropicamide to a diameter of 7–8 mm. Pupil diameter was measured by an observer (LZ) by means of a ruler and a magnifying lens and stored for each tested eye. The cornea was anesthetized with 1% Dicaine. MfERGs were recorded bipolarly between an active electrode (Dawson Trick Litzkow (DTL) bipolar contact electrode) and a reference electrode (Ag/AgCl electrode placed on the correspondent temporal side of the frontal lobe). A small Ag/AgCl skin ground electrode was placed at the center of the forehead. Interelectrode resistance was less than 3 KOhms. Binocular MfERG recording was preferred for helping subjects to have a stable target fixation. Eyes that did exhaustively meet the inclusion criteria were selected from each patient. The signal was amplified (gain 100.000) and filtered (band pass 1–100 Hz) by BM 6000 (Biomedica Mangoni, Pisa, Italy). After automatic rejection of artifacts (by VERIS Clinic™ 4.9 software), the first-order kernel response, K1, was examined.

#### 2.2.1. Ring Analysis

MfERG ring analysis was selected to differentiate changes of the bioelectrical responses of the central foveal regions with respect to the more eccentric retinal areas in the macular region. We analyzed the averaged response obtained from three concentric annular retinal regions (rings) centered on the fovea: 0 to 2.5° (ring 1, R1), from 2.5 to 5° (ring 2, R2), from 5 to 10° (ring 3, R3). We also analyzed the responses from unified rings enclosing responses derived from the total area from 0–5° (R1 + R2) and from the fovea up to 10°(R1 + R2 + R3). For each obtained averaged response, we evaluated the amplitude densities (RAD, expressed in nanovolt/degree^2^) between the first negative peak, N1, and the first positive peak, P1, and the implicit time (IT) of the first positive peak (P1).

#### 2.2.2. Sector Analysis

MfERG sector analysis was selected to differentiate changes of the bioelectrical responses of the central macular region in 4 quadrants: inferior (I), nasal (N), superior (S), and temporal (T). We considered isolated and combined responses from the foveal center to external areas (sector 1, S1: 0–2.5°; sector 2, S2: 0–5°; and sector 3, S3: 0–10°) and S1 + S2 and S1 + S2 + S3, respectively. A similar analysis was recently adopted to study retinal functional changes in a hereditary ocular pathology [26].

### 2.3. Laboratory Measurements

Plasma glucose concentrations were measured by the hexokinase method (Modular P Analyzer, Roche). The intra-assay coefficient of variation (CV) was 1.1%, and interassay CV was 1.9%. The sensitivity of the method was 2 mg/dl (0.11 mmol/l). HbA1c was analyzed by high-performance liquid chromatography (VARIANT 2; BioRad Laboratories, Munich, Germany), with intra- and interassay CV of 0.46–0.77 and 0.69–0.91%, respectively. Plasma total cholesterol, high-density lipoprotein cholesterol (HDL chol), and low-density lipoprotein cholesterol (LDL chol) were analyzed with a colorimetric enzymatic method (CHOD-PAP, Roche Diagnostics). The intra-assay CV was 1%, and the interassay CV was 2.7%. The sensitivity of the method was 0.08 mmol/l. Plasma triglycerides were analyzed with a colorimetric enzymatic method (GPO-PAP, Roche Diagnostics). The intra-assay CV was 1.5%, and the interassay CV was 2.4%. The sensitivity of the method was 0.05 mmol/l. Urinary albumin was determined by the Tina-quant immunoturbidimetric assay (Cobas, Roche Diagnostic, Indianapolis, IN) and urinary creatinine by an enzymatic colorimetric test (Beckmann Coulter, California, USA). C underwent an oral glucose tolerance test, to exclude diabetes and impaired glucose tolerance.

## 3. Statistical Analysis

The main statistical analysis aimed at assessing the difference among T1DM and C groups in terms of MfERG RADs and MUNE. Thus, a general linear model was applied, allowing to adjust for eventual demographic, anthropometric, and metabolic differences.

Correlations among interval variables were measured through Pearson's index, after appropriate log-transformation when necessary. In order to verify the robustness of correlations, influence statistics (standardized DfBetas) were computed and, in case of outliers, correlations were computed after their elimination.

## 4. Results and Discussion

**Figure 1:**
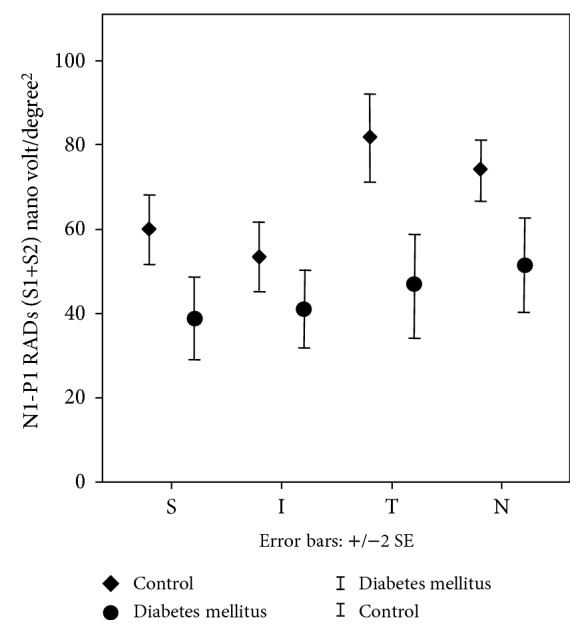
Combined sector analysis of mean MfERG RADs from S1 + S2 (0–5°) in the superior, temporal, and nasal sectors showed significantly reduced values in T1DM subjects vs. C (*p* < 0.05). There was also a reduced but not statistically significant RAD value from the inferior sector (0.055). In addition, significant sector X group interaction was found (*F*(3,81) = 6.07; *p* = 0.001), since the difference between DM and C was larger in the temporal and nasal sectors with respect to the superior and inferior sectors (consistently, *p* < 0.005) N1-P1 RADs are defined as amplitude densities between the first negative peak, N1, and the first positive peak, P1. S: superior; I: inferior; T: temporal; N: nasal macular quadrants.

Clinical and laboratory characteristics of the diabetic patients and of the C group are reported in Table 1. Seven out of 20 patients had mild NPDR. The T1DM and C groups were not different, except for body mass index (BMI), fasting glucose, and HDL cholesterol levels. All patients had an MRC score of 60/60 at neurological examination indicating full strength. From the MfERG analysis, the mean MfERG RADs of R1 (0–2.5°), R2 (2.5–5°), and R3 (5–10°) differed significantly between the C and diabetic groups (*p* < 0.01). Combined sector analysis of mean MfERG RADs from S1 + S2 (0–5°) and S1 + S2 + S3 (0–10°) in superior, temporal and nasal sectors showed significantly reduced values in T1DM subjects vs. C (*p* < 0.05). There was also a reduced, but not statistically significant, RAD value from the inferior sector (0.055). In addition, significant sector X group interaction was found (*F*(3,81) = 6.07; *p* = 0.001), since the difference between DM and C was larger in the temporal and nasal sectors with respect to the superior and inferior sectors (consistently, *p* < 0.005) (Figure 1). For the IT parameter, no significant differences were found between the C and diabetic groups. No significant differences were found between noDR and NPDR patients for both RADs and ITs.

**Figure 2:**
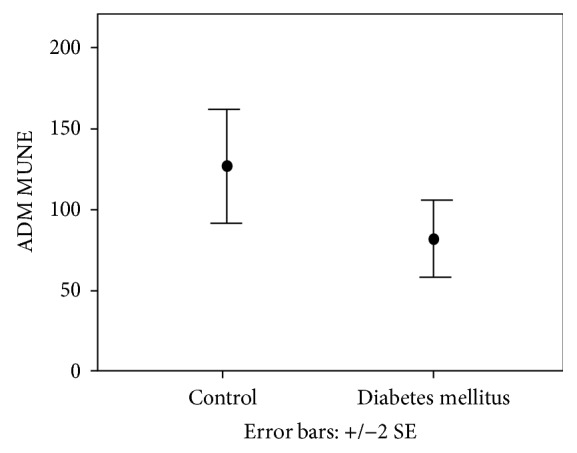
Motor unit number estimation (MUNE) (mean ± SEM) of abductor digiti minimi (ADM) in the diabetic groups and controls. ADM MUNE was significantly decreased in T1DM vs. C (*p* < 0.05).

**Figure 3:**
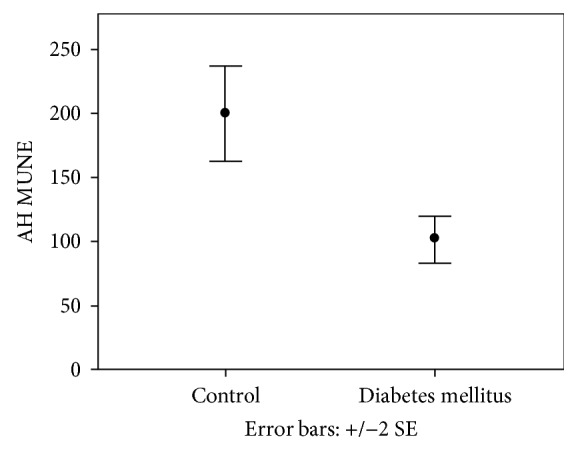
Motor unit number estimation (MUNE) (mean ± SEM) of abductor hallux (AH) in the diabetic groups and controls. AH MUNE was significantly decreased in T1DM vs. C (*p* < 0.001).

No abnormalities of conventional NCS were found in any subject. The number of motor units was significantly decreased in both the lower and upper limbs in T1DM vs. C (ADM MUNE: 82.55 ± 54.37 vs. 126.96 ± 65.85, *p* = 0.039; AH MUNE 101.87 ± 41.09 vs. 199.90 ± 69.81, *p* < 0.001), while AH SMUP was significantly increased in T1DM vs. C (0.55 ± 0.17 vs. 0.35 ± 0.13 *μ*V/msec, *p* = 0.001) (Figures 2 and 3).

Since BMI and HDL chol were significantly different among the two groups, we added these variables as covariates in the previous analyses and the patterns remained stable.

**Figure 4:**
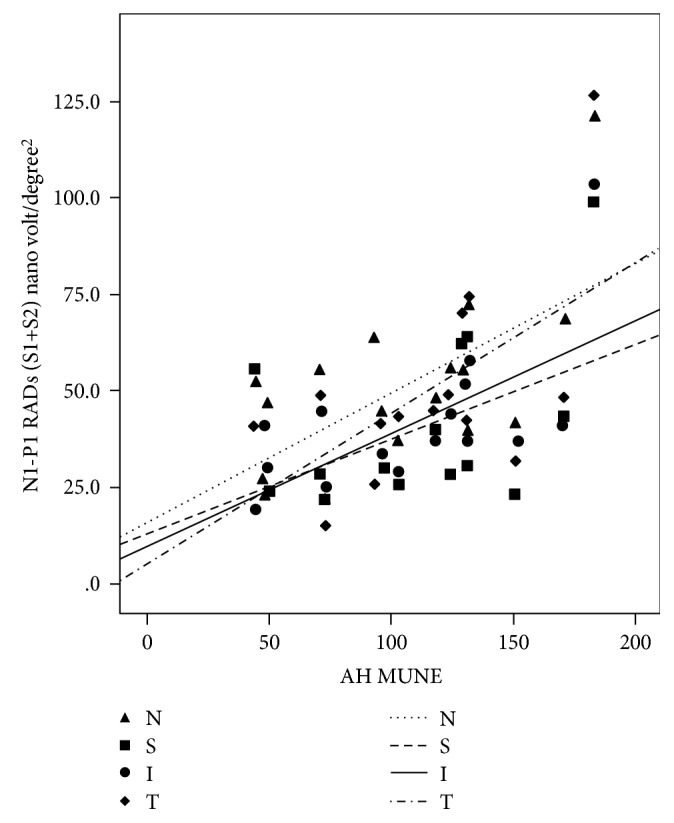
Scatter plot between MfERG RADS of S1 + S2 of the N (nasal), S (superior), I (inferior), and T (temporal) sectors and motor unit number estimation of the abductor hallux (AH MUNE) in type 1 DM patients. A positive correlation between the mean MfERG RADs of the four quadrants (S, I, T, and N) and AH MUNE (*r* = 0.50, *p* = 0.041; *r* = 0.64, *p* = 0.005; *r* = 0.64, *p* = 0.006; and *r* = 0.61, *p* = 0.010, respectively) was observed in T1DM patients. Since such correlations could be significantly affected by outliers, influence statistics (standardized DfBetas) were computed. After eliminating cases with values higher than the cutoff (2/sqrt(*n*)), *r* correlations resulted similar (*r* = 0.49, *p* = 0.065; *r* = 0.64, *p* = 0.015; *r* = 0.64, *p* = 0.013; and *r* = 0.52, *p* = 0.048, respectively).

A positive correlation between the mean MfERG RADs of S1 + S2 of the four sectors (S, I, T, and N) and AH MUNE (*r* = 0.50, *p* = 0.041; *r* = 0.64, *p* = 0.005; *r* = 0.64, *p* = 0.006; and *r* = 0.61, *p* = 0.010, respectively) was observed in T1DM patients (Figure 4). Since such correlations could be significantly affected by outliers, influence statistics (standardized DfBetas) were computed. After eliminating cases with values higher than the cutoff (2/sqrt(*n*)), *r* correlations resulted similar (*r* = 0.49, *p* = 0.065; *r* = 0.64, *p* = 0.015; *r* = 0.64, *p* = 0.013; and *r* = 0.52, *p* = 0.048, respectively).

We did not observe a significant correlation between the HbA1c and metabolic parameters and retinal macular function or MUNE (consistently, *p* > 0.2).

In this study, the diabetic subjects were highly selected without DPN, both clinically and after routine electrophysiological examination. Moreover, the good glycemic control and the absence of other microvascular complications and comorbidities allowed to perform these evaluations in the absence of confounding factors. Despite this, we observed a significant reduction in the number of motor units of both the ADM muscle (upper limb) and, more pronounced, the AH muscle (lower limb), consistent with length-dependent neurodegeneration expected in diabetes. The MUNE technique is increasingly used in studies on amyotrophic lateral sclerosis, but it is more rarely used in other peripheral nerve disorders, including diabetic neuropathy [6, 27–30].

Our findings imply that loss of motor units occurs early in the neurodegenerative process in diabetic patients and MUNE can detect motor unit abnormalities, even in the absence of signs of diabetic neuropathy at clinical exam and conventional NCS. Similarly to what is observed for epidermal nerve fiber densities, the dying back of motor nerve terminals occurs early and in the absence of clinical sensory-motor symptoms and signs in DM [31]. We also observed an increase in AH SMUP in the diabetic subjects compared to the control group. In diabetic peripheral neuropathy, a process of chronic denervation, the germination of small intact motor units can lead to larger SMUPs. We know that a motor unit includes a single motor neuron and the group of muscle fibers that it innervates. The increase in the average size of motor units suggests a preferential loss of smaller motor units or the enlargement of these units by compensatory germination [32]. The size of the motor units can also increase with axonal sprouting, which reinnervates the denervated muscle fibers to compensate for the loss of adjacent functional motor units [32–34]. The findings of concurrent AH SMUP enlargement can account for the existence of a similar process already ongoing in these asymptomatic diabetic patients. The only electrophysiological parameter currently used to describe the neuronal loss in neuropathies is the amplitude of distal cMAP, but this parameter remains into normal range until the reinnervation process is effective. Only when a marked loss of the motor units is reached, the force production will decrease in parallel, resulting in the reduction of cMAP amplitude in conventional NSC studies and muscle weakness as described in diabetic patients with more advanced peripheral neuropathy. Then, MUNE may represent an early noninvasive marker of a subclinical DNP. Such a marker has potential therapeutic implications, allowing an early treatment approach for DPN, when the probability of regeneration of sensorimotor fibers is still good [6].

We also showed that multifocal electroretinogram is a valuable tool in order to examine local neuroretinal dysfunction in T1DM with or without diabetic retinopathy. Therefore, it is able to detect functionally the most vulnerable areas, even in the absence of ophthalmoscopic early signs of retinal abnormalities. MfERG allows for the simultaneous recording of the activity of bipolar cells with small contributions from photoreceptors from different areas of the retina [35]. In diabetic population studies, the use of MfERG [36–39] identified significant abnormalities in retinal function, characterized by increased peak latencies and/or reduced amplitudes, suggesting a compromised inner retinal function, secondary to neuronal transmission alterations [40]. The topographic mapping of neuroretinal dysfunction by MfERG has been shown to be predictive of the onset of DR [36] and is able to detect these abnormalities earlier than morphological studies [41, 42]. Recently, in adolescents with T1DM and noDR, an early alteration of the inner retina, confirmed also by a delay of IT in MfERG recordings, has been observed [43]. The nasal retina had abnormal IT compared with the temporal retina, whereas alteration of the amplitude parameter was more evident in the temporal retina in T1DM adolescents [44]. Moreover, Holm and Adrian [45] described that the nasal area of the macula, where there is a higher density of cones and ganglion cells, was more vulnerable to neurodegenerative processes than the temporal region, showing a lower amplitude and longer implicit time in this specific area with the MfERG analysis. The innovative MfERG sector analysis allowed to identify the specific retinal areas of neuroretinal damage that in our group of patients are mainly represented by the nasal and temporal sectors, anatomically crucial regions for the function of collector cells to the small axons of the optic nerve forming the papillomacular bundle. The neurodegenerative theory, for which the photoreceptors are involved early in the course of diabetes in patients, has been also supported by recent *in vivo* studies using adaptive optics ophthalmoscopy [46]. The authors have shown that early pathological disruption of the parafoveal cone mosaic in patients with type 1 diabetes, even before any sign of diabetic retinopathy, was found on fundoscopy. Furthermore, through the use of spectral domain optical coherence tomography (SD-OCT) analysis, we recently observed an increased macular thickness of the inner nuclear layer (INL) and a decrease in the retinal nerve fiber layer (RNFL) thickness in the nasal quadrant of the macular area in T1DM persons with noDR and NPDR, compared to healthy groups. In order to analyze the role of glycemic control on the neuroretina, we also found that only glycemic variability was associated with abnormalities of these specific retinal layers, while no association was observed with HbA1c [14]. Finally, we studied the relationship between functional changes in the neuroretina and early signs of peripheral neuropathic damage. We have found that motor unit loss was associated with the amplitude densities' reduction in all four macular sectors, from the foveal center up to the 5° external areas. At the moment, the available evidence on the link between retinal neurodegeneration and diabetic neuropathy is scanty; it is related exclusively to morphological evidence and identifiable especially in DM1 or DM2 patients already affected by peripheral neuropathic damage diagnosed with standard examinations [47–51]. Recently, in a prospective study, defining longitudinal alterations to the RNFL thickness of the optic nerve head in individuals with DM1, with and without DPN, patients with DPN showed a progressive global RNFL thinning, especially in the superior quadrant. Therefore, it is possible to hypothesize common pathways for retinal and peripheral neurodegeneration that are independent of DPN risk factors [51]. Polyol pathway activation, hexosamine pathway and protein kinase C (PKC) isoform activation, and accumulation of advanced glycation end products (AGEs) resulting in imbalance of the mitochondrial redox state and in excess formation reactive oxygen species may represent common mechanisms to neuroretinal and peripheral neurodegeneration. Furthermore, a key role of the glial component has been described in the early stages of both neuronal and neuroretinal damages [14, 52, 53]. Despite promising evidence linking morphological alterations of the neuroretina to the overt presence of diabetic neuropathy, a functional analytical approach is required to identify and characterize initial stages of neuropathic damage. Very few studies have investigated the role of early outer retinal deficit at the base of retinal neurodegeneration [46], and, to our knowledge, this is the first work that allowed to observe a relationship between this dysfunction at the early peripheral neuropathic damage. In light of this, our result of an association between neuroretinal (photoreceptors and bipolar cells) dysfunction and subclinical damage of the peripheral nerve in asymptomatic diabetic subjects seems particularly interesting. MfERG recording could represent an accessible, noninvasive, and well-tolerated tool in the detection also of neuronal damage in diabetic patients. However, our study has some limitations: small sample size and the presence in the diabetic population of subjects with diabetic vascular retinopathy, although not proliferating and of a very mild degree, the lack of an association with standardized methods of early DN diagnosis, such as skin biopsy. The inclusion of a cohort of patients with overt DN or a longitudinal observation could allow to understand the associative link between the two neurodegeneration processes. Nevertheless, we believe that the identification of these potential markers of very early neuropathic damage in diabetes (retinal neurodegeneration and morphofunctional alterations of the motor unit) is an important point of strength of our study. Our findings, therefore, strongly support a new vision of the neuropathic damage in diabetes, as an overall neuronal damage.

## 5. Conclusions

In conclusion, motor unit loss and neuroretinal dysfunction are already present in T1DM patients without DPN. The relationship between neuroretinal dysfunction and early peripheral motor unit decline supports the hypothesis that the neuroretina is a potential “window” onto the early neurogenic process, in diabetes.

## Figures and Tables

**Table 1:** Clinical and laboratory characteristics of controls (C) and subjects with diabetes (T1DM), mean (SD).

	C *n* = 14	T1DM *n* = 20	*p* value
Gender (M/W)	5/9	9/11	*p* = 0.588
Age (yrs)	39.07 (14.4)	42.3 (12.4)	*p* = 0.721
Diabetes duration (yrs)	—	17.9 (9.5)	—
BMI (kg/m^2^)	22.5 (±2)	24.9 (± 2.5)	*p* < 0.001
Glycemia (mmol/l)	4.9 (0.6)	8.9 (1.4)	*p* < 0.0.001
HbA1c (%) (mmol/mol)	—	7.5 (0.8) 58 (15)	—
Tot chol (mmol/l)	4.0 (0.6)	4.3 (0.44)	*p* = 0.089
HDL chol (mmol/l)	1.7 (0.2)	1.5 (0.2)	*p* = 0.002
Trigl (mmol/l)	0.8 (0.1)	0.75 (0.1)	*p* = 0.08
Microalb/creat (mg/gr)	—	7.2 (5.2)	—
Sural nerve (lateral malleolus)	—	Distal SNAP amp (*μ*V) 13.4 (±4.3)	—
		SCV (ms) 56.4 (±6.9)	—
Tibial nerve (AH)^∗^	—	Distal CMAP latency (ms) 3.2 (±0.4)	—
		Distal cMAP amplitude (*μ*V) 11.9 (±4.6)	—
		MCV (ms) 47.3 (±4.5)	—
Ulnar nerve (ADM)^∗^	—	Distal CMAP latency (ms) 2.2 (±1.7)	—
		Distal cMAP amplitude (*μ*V) 9.8 (±1.7)	—
		MCV (ms) 59.7 (±5.3)	—

^∗^No patient showed abnormalities (temporal dispersion or conduction block) in intermediate and proximal nerve segments. M: men; W: women; BMI: body mass index; HbA1c: hemoglobin glycated; Tot chol: total cholesterol; HDL chol: high-density lipoprotein cholesterol; Trigl: triglycerides; Microalb/creat: microalbuminuria/creatininuria; AH: abductor hallux; ADM: abductor digiti minimi; SNAP: sensory nerve action potential; MCV: motor conduction velocity; SCV: sensory conduction velocity; *t*-test. Statistical significance *p* < 0.05.

## Data Availability

The data used to support the findings of this study are available from the corresponding author upon request.
